# Generalist–specialist trade-off during thermal acclimation

**DOI:** 10.1098/rsos.140251

**Published:** 2015-01-21

**Authors:** Frank Seebacher, Varlérie Ducret, Alexander G. Little, Bart Adriaenssens

**Affiliations:** 1 School of Biological Sciences, University of Sydney, New South Wales 2006, Australia; 2 Department of Ecology and Evolution, UNIL Sorge, Le Biophore, Université de Lausanne, 1015 Lausanne, Switzerland; 3 Institute of Biodiversity, Animal Health and Comparative Medicine, University of Glasgow, Glasgow G12 8QQ, UK

**Keywords:** performance curves, locomotor performance, performance breadth, individual variation, environmental variability

## Abstract

The shape of performance curves and their plasticity define how individuals and populations respond to environmental variability. In theory, maximum performance decreases with an increase in performance breadth. However, reversible acclimation may counteract this generalist–specialist trade-off, because performance optima track environmental conditions so that there is no benefit of generalist phenotypes. We tested this hypothesis by acclimating individual mosquitofish (*Gambusia holbrooki*) to cool and warm temperatures consecutively and measuring performance curves of swimming performance after each acclimation treatment. Individuals from the same population differed significantly in performance maxima, performance breadth and the capacity for acclimation. As predicted, acclimation resulted in a shift of the temperature at which maximal performance occurred. Within acclimation treatments, there was a significant generalist–specialist trade-off in responses to acute temperature change. Surprisingly, however, there was also a trade-off across acclimation treatments, and animals with greater capacity for cold acclimation had lower performance maxima under warm conditions. Hence, cold acclimation may be viewed as a generalist strategy that extends performance breadth at the colder seasons, but comes at the cost of reduced performance at the warmer time of year. Acclimation therefore does not counteract a generalist–specialist trade-off and, at least in mosquitofish, the trade-off seems to be a system property that persists despite phenotypic plasticity.

## 2. Introduction

Persistence of populations in variable environments depends on how well individuals can withstand changing abiotic conditions [1,2]. Responses of individuals to changing environmental conditions are characterized by performance curves that describe changes in a physiological rate with an acute change in an environmental condition, such as temperature [1,3]. Performance curves typically have a maximum (or mode) that occurs over a narrow range of conditions, with performance decreasing at higher and lower values of the environmental variable [1,3,4]. Performance breadth may be defined as the range of environmental values over which performance is greater than a given proportion of maximum (e.g. greater than 80 or 90% of maximum [1,3,5,6]).

Reversible acclimation within individuals can lead to compensation for changing environmental conditions and thereby increase resilience to environmental change [4,7,8]. Alternatively, individuals may possess a generalist phenotype that reduces sensitivity to environmental change, but also leads to a decrease in maximal performance compared with individuals that are specialized to stable conditions [1,5,6]. This specialist–generalist trade-off is thought to arise because physiological and biochemical processes can be maximized only at one particular set of environmental conditions [9]. However, acclimation could negate the specialist–generalist trade-off because, in the case of perfect acclimation or compensation, performance curves would shift so that performance optima coincide with prevailing environmental conditions [4,7,8]. The effect of acclimation on the shape of performance curves and the specialist–generalist trade-off is unresolved, however, and our aim was to determine how thermal acclimation influences locomotor performance curves in mosquitofish, *Gambusia holbrooki*. We consider differences between individuals from the same populations, but similar arguments can be applied also to differences between populations or species [1]

**Figure 1.**
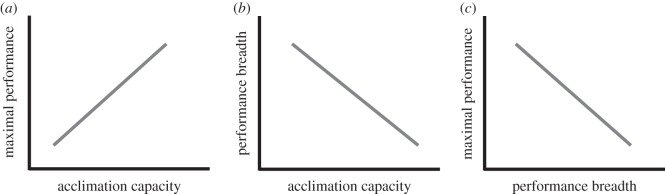
Graphic representation of the hypotheses tested. Acclimation can shift reaction norms so that performance optima coincide with mean environmental conditions. Generalist phenotypes with broader performance breadth and lower performance maxima would therefore not be advantageous in animals that have the capacity for acclimation. We therefore predict that, across acclimation conditions, maximum performance will increase (*a*) and performance breadth will decrease (*b*) with increasing acclimation capacity. However, in response to acute changes in environmental conditions that occur too fast to allow for acclimation of physiological systems, physiological and biochemical constraints will cause a generalist–specialist trade-off (*c*).

Thermal acclimation involves re-modelling of physiological processes, such as metabolism and muscle function [4,7,10,11], so that the temperature at which maximum performance occurs tracks changes in mean environmental conditions. Acclimation typically takes several weeks to establish [12–14]. Hence, acclimation is not responsive to short-term (e.g. daily) thermal variation, but it can be effective in compensating for longer term variation (e.g. seasonal). In individuals that have the capacity for acclimation, generalist phenotypes would be disadvantageous because thermal optima track environmental conditions, and selection for increased performance breadth at the cost of reduced performance maxima would not increase fitness. We predict, therefore, that animals with greater capacity for acclimation will also have greater performance maxima (figure 1*a*) and reduced performance breadth (figure 1*b*). Alternatively, the time frame of thermal change may be important, because acclimation does not compensate for acute changes in temperature. Hence, we predict that in chronically stable conditions, such as within seasons or temperature treatments, biochemical and physiological constraints [9] will cause variation in performance breadth and maximal performance between individuals along with the trade-off between these strategies (figure 1*c*). However, given time to acclimate physiological and biochemical systems the trade-off should disappear across chronic conditions.

To test these predictions, we acclimated mosquitofish (*G. holbrooki*) first to a low temperature (three weeks at 20°C), and then measured the acute thermal sensitivity of swimming performance across a range of temperatures. The same individuals were then acclimated to a high (28°C) temperature followed by measurements of acute thermal sensitivity of sustained swimming performance.

## 3. Material and methods

### 3.1 Study animals and acclimation treatments

Juvenile (less than 16 mm standard length) mosquitofish, *G. holbrooki*, of both sexes were collected from Manly Dam, Sydney, Australia (33°78^′^ S; 151°26^′^ E) using a large dip-net and transported to the University of Sydney. Cohorts of fish of the same age school together and are therefore easily identified. In the experiments, we used fish of the same age and collected at the same time in summer (February) only, so that all experimental fish would have developed under the same environmental conditions. For two weeks after collection, schools of fish were housed in plastic tanks (400×270×350 mm) at 24°C, which corresponds approximately to the natural water temperature at that time of year [15], at a stocking density of one to two fish per litre. Each tank contained an activated carbon filter. Fish were exposed to a 14 L : 10 D photoperiod and fed ad libitum daily with fish food flakes (Optimum Freshwater Flakes, New Life Spectrum, Homestead, FL, USA).

After this habituation period, fish were placed singly into individual containers (1 l plastic cylinders, with vertical slits in the side) that were suspended in plastic tanks (6–12 individual containers per tank, 400×270×350 mm). This arrangement permitted us to follow individual fish over the length of the experiment, while still permitting visual and olfactory contact between fish. After fish were separated, tank temperatures were changed gradually over 3 days to reach the desired acclimation temperatures of 20°C or 28°C. There were three tanks per acclimation treatment, each containing 6–12 fish in their individual enclosures. Fish were kept for three weeks at their respective acclimation temperatures before measurements were taken. All fish were first acclimated to 20°C, followed by acclimation to 28°C and the same procedures were used for each acclimation treatment. Data from rainbow trout indicate that thermal acclimation is reversible so that the order of acclimation treatment is unlikely to have a strong effect [12]. The acclimation temperatures were chosen because they represent the upper and lower temperatures that would have been experienced naturally by that cohort of fish [15]. Each individual fish was exposed to both acclimation conditions consecutively, and swimming performance was measured after each acclimation treatment. All fish reached maturity by the time the first measurements of swimming performance were taken after the first acclimation treatment, and mean standard length was 23.3±0.43 (s.e.) mm and mean mass was 0.13±0.016 (s.e.) g. Note that because fish matured in isolation, none of the females were pregnant.

### 3.2 Swimming performance

Following each acclimation treatment, we measured sustained swimming performance (*U*_crit_ [16]) of male (*n*=20) and female (*n*=28) fish. We measured swimming performance at the acclimation temperatures (20°C and 28°C) plus at a higher temperature (32°C) to measure performance at a temperature higher than that at which the maximum occurs; note that it was unlikely that maxima would occur below 20°C [17] so that we did not measure swimming performance below 20°C. We chose 32°C rather than a higher temperature to avoid exposing fish to damaging or lethal temperatures, which would have been unacceptable ethically and which would have precluded us from using fish in the second acclimation treatment; in the event, maximal performance of some fish occurred at temperatures higher than 32°C so that we could not use these data for some of the analyses (see below). We chose sustained swimming performance as the response measure because it is a whole-animal performance trait that integrates numerous underlying physiological processes, and it is closely related to fitness [18–20]. Sample sizes for males are somewhat lower than for females because we had to discard some data as a result of problems with pumps.

*U*_crit_ was measured in a swimming flume consisting of a 150×50 mm clear Perspex cylinder tightly fitted over the intake end of a submersible inline pump (12 V DC, iL500, Rule, Hertfordshire, UK). A plastic grid separated the Perspex swimming flume from the pump and a bundle of hollow straws at the inlet helped maintain laminar flow; the flume was contained in a plastic tank (645×423×276 mm). We used a variable power source (MP3090, Powertech, Sydney, New South Wales, Australia) to adjust the flow speed. To each pump, we connected a flow meter (6710 M, DigiFlow, Taiwan) that provided flow rate in real time. Fish were swum initially for 20 min at 6 cm s^−1^, when flow velocity in the flume was increased in steps (*U*_*i*_) of 2 cm s^−1^ every 5 min (*T*_*i*_). *U*_crit_ was determined as *U*_crit_=*U*_*f*_+*T*_*f*_/*T*_*i*_×*U*_*i*_, where *U*_*f*_ is the highest speed maintained for an entire interval (*T*_*i*_=5 min), *T*_*f*_ is the time until exhaustion at the final speed interval. A fish was defined to be exhausted when it could no longer keep its position in the water column after two chances; that is, when the fish first fell back on the plastic grid, water flow was reduced immediately until the fish swam again and then increased again to the previous velocity. The next time the fish fell back, the trial was ended. We expressed swimming performance as body lengths per second (BL s^−1^).

### 3.3 Statistical analysis

We used general linear mixed models (LMM) to analyse how acclimation, test temperature and their interaction affected *U*_crit_. All LMM analyses were completed using package lme4 in R [21,22]. We were interested in both how temperature treatments affected overall group mean performance and how individual responses to temperature treatments differed. LMM jointly accounts for repeated measures across individuals while enabling hypothesis testing about overall group mean effects (fixed effects) and individual differences in these effects (random effects). Test temperature was added both as a linear effect (TT) and a quadratic effect (TT^2^) to account for the nonlinear, peaked, shape of the thermal performance curve [3]. These shape components of the thermal performance curve estimated, respectively, the linear slope and the curvature of the thermal performance curve. Larger linear slope estimates indicate higher optimal temperatures, and more negative estimates for curvature indicate greater thermal sensitivity with more ‘peaked’ performance curves. Test temperature was expressed as degrees Celsius deviation from 28°C in order to allow estimation of the slope and curvature closest to the intermediate temperature. Before analysis, *U*_crit_ in body lengths per second was transformed to units standard deviation from the mean.

Overall group mean effects were assessed by fitting the following model (table 1, model 1):

$$U_{\mathrm{crit}(ij)}=\beta_{0}+\beta_{1}\mathrm{sex}+\beta_{2}\text{TT}+\beta_{3}{\text{TT}}^{2}+\beta_{4}\text{AT}+\beta_{5}{\text{TT}}^{*}\text{AT}+\beta_{6}{\text{TT}}^{2}{\;}^{*}\text{AT}+{\text{ID}}_{\left(0j\right)}+{\text{error}}_{\left(0ij\right)},$$

in which ID_(0*j*)_ represents the individual-specific random effect, or individual differences in *U*_crit_ across all temperature treatments. As fixed covariates, we estimated *β*_1_ as the overall mean difference in *U*_crit_ between males and females; *β*_2_ is the slope of the thermal performance curve, that is the location of the optimal temperature in relation to 28°C; *β*_3_ as the curvature of the thermal performance curve. Additional fixed covariates estimated the effect of acclimation (AT) on the thermal performance curve: a vertical shift of the thermal performance curve (*β*_4_), a change in linear slope (*β*_5_) and curvature (*β*_6_). To conclude, *β*_0_ represents the overall intercept and error_(0*ij*)_ the residual error. Significance levels and confidence intervals of the fixed effects were calculated using maximum-likelihood estimation and the Satterthwaite method for estimation of degrees of freedom (package lmerTest in R [23]).

**Table 1.** Significance tests of fixed covariates as tested within the most simple random effect model (model 1). Probabilities for fixed effects were calculated using the lmerTest package in R and Satterthwaite's approximation for degrees of freedom.

	*β*	s.e.	d.f.	*t*	*p*-value
(intercept)	0.49	0.12	209.39	3.88	<0.0001
AT	0.27	0.15	239.97	1.77	0.077
TT	−0.076	0.023	239.97	−3.34	0.001
TT^2^	−0.024	0.0042	239.97	−5.65	<0.0001
sex	−0.33	0.12	47.99	−2.68	0.001
AT : TT	0.15	0.032	239.97	4.66	<0.0001
AT : TT^2^	0.011	0.0059	239.97	1.82	0.070

Differences between individuals in their responses to acclimation and test temperatures were assessed by gradually building more complex models by adding random effects (see table 2 for random effect specification). Likelihood ratio tests were used to compare more complex models to their simpler variant using the restricted maximum-likelihood method. The first three more complex models assessed individual differences in thermal sensitivity independent of acclimation temperatures. First, a linear random slope for test temperature was nested within individuals to account for individual differences in the optimal temperature (model 2). Similarly, model 3 accounted for individual differences in the curvature of the thermal performance curve by adding the quadratic effect of test temperature nested within individuals to model 1. Model 4 assessed the joint importance of individual differences in both linear slopes and curvature of the thermal performance curves. We then assessed the importance of individual differences in the effect of acclimation on performance. Differences in how individuals adjusted average performance in response to acclimation were accounted for in model 5 (‘vertical shift’ of thermal performance curve). Additional models accounted also for how individuals differed in acclimation-caused changes to their optimal performance temperature (i.e. linear slope; model 6), its curvature (model 7) and joint changes in slope and curvature (model 8).

**Table 2.** Different random effect models used and their comparisons with REML likelihood ratio tests. Comparisons between different models (M) are shown; ID, individual fish identity; TT, test temperature linear; TT^2^, test temperature quadratic; AT, acclimation temperature; AIC, Akaike Information Criterion; Llike, loglikelihood; ref M, reference model; a colon ‘:’ indicates an interaction, and ‘|’ indicates a conditional relationship. All models contained the full fixed effect structure as described in the main text alongside the random structure.

M	random terms	ref M	M d.f.	AIC	Llike	*χ*^2^	d.f.	*p*-value
1	(1|ID)		9	692.8	−337.4			
2	(TT|ID)	1	11	692.3	−335.2	4.5	2	0.11
3	(TT^2^|ID)	1	11	694.0	−336.0	2.8	2	0.25
4	((TT+TT^2^)|ID)	1	14	698.2	−335.1	4.6	5	0.47
5	(AT|ID)	1	11	681.1	−329.6	15.7	2	0.0004
6	(((TT:AT)+AT)|ID)	5	18	679.4	−321.7	15.8	7	0.027
7	(((TT^2^:AT)+AT|ID)	5	18	682.9	−323.5	12.2	7	0.094
8	(((TT:AT)+(TT^2^:AT)+AT)|ID	1,5,6,7	29	650.8	−296.4	>50.0	>11	<0.0001

We further analysed the quadratic performance curves to test the hypothesis that acclimation influences the relationship between performance breadth and maximum performance. We defined performance breadth as the temperature range within which swimming performance was 80% of the maximum performance or greater. We fitted quadratic curves to the swimming data from each fish (in Prism 5, GraphPad Software Inc., CA, USA) and calculated the maximum value by differentiating and determining *f*′(*x*)=0. We calculated the temperatures to the left and right of the maximum at which 80% of performance occurred by subtracting 80% of the maximum and taking the quadratic roots using the quadratic formula. This analysis was limited to those animals in which performance was greatest at temperatures of less than 32°C, i.e. within the range of our experimental test temperatures, because otherwise we could not determine the maximum of the quadratic equation reliably. We determined performance breadth separately for warm and cold acclimation (*n*=34 for cold acclimation and 28 for warm acclimation). However, we did not distinguish between males and females to maintain adequate sample sizes for the regression analyses.

We determined the capacity for acclimation or temperature compensation as follows:

$$\text{acclimation capacity}=1-\left(\frac{P_{28}-P_{20}}{P_{28}+P_{20}/2}\right),$$

where *P*_28_ denotes performance of fish acclimated to 28°C and measured at 28°C, and *P*_20_ denotes performance of fish acclimated to 20°C and measured at 20°C. Perfect temperature compensation would mean that performance of fish acclimated to 20°C and measured at 20°C would be identical to that of fish acclimated to 28°C and measured at 28°C; in this case the acclimation capacity would be 1. The lower the capacity for acclimation to cold temperatures (i.e. $P_{28}-P_{20}\to P_{28}$), the smaller the value for acclimation capacity.

We analysed the relationship between compensation and performance breadth and maxima, and also between maximum performance and performance breadth by ordinary least-squares regression analysis [24].

## 4. Results

### 4.1 Fixed effects

**Figure 2.**
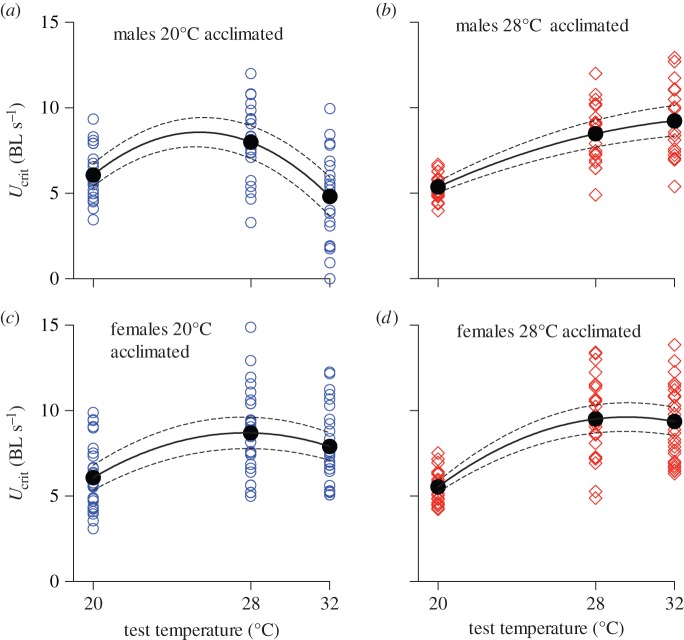
Mean performance curves (black lines and filled circles; dashed lines indicate 95% CIs) of sustained swimming (*U*_crit_) in mosquitofish. The temperature at which swimming performance was maximal differed between acclimation treatments (20°C=blue circles (*a*,*c*); 28°C=red diamonds (*b*,*d*)), and males (*a*,*b*) had overall lower performance than females (*c*,*d*). There was considerable variation between individuals (individual circles), which manifested both in the curvature of the performance curve and the temperature at maximal performance. Individual variation resulted from differences between individuals in their capacity for acclimation.

Both linear and curvature components of the thermal performance curve were significant (table 1 and figure 2). Acclimation to 28°C resulted in a greater sustained swimming performance across all test temperatures when compared with acclimation to 20°C (AT effect, ‘vertical shift’, table 1 and figure 2). Acclimation to 28°C also increased the temperature at which maximal sustained swimming occurred (AT*TT effect, table 1), and showed a non-significant (*p*=0.07) tendency to flatten the concave curvature of the thermal performance curve (AT*TT^2^ effect, table 1). Hence, 26% of cold acclimated fish and 41% of warm acclimated fish had performance maxima at temperatures above 32°C. In addition, males exhibited a lower sustained swimming speed than females (sex effect, table 1 and figure 2).

### 4.2 Individual differences

The model that best described the individual differences in thermal responses was the model that accounted for individual variation in acclimation-caused changes in optimal temperature, curvature and vertical shifts of the thermal performance curve (model 8, table 2). However, individual differences in thermal sensitivity were solely influenced by variation in the ability to respond to acclimation (no significant difference between models 1 and 4).

**Figure 3.**
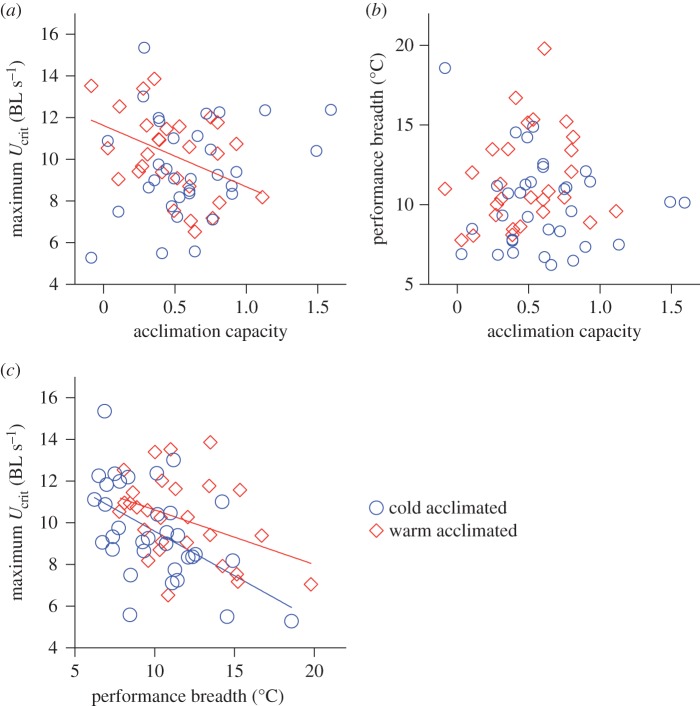
Relationship between acclimation and performance curves. Maximal performance during warm acclimation (28°C; red diamonds), but not during cold acclimation (18°C; blue circles), decreased with increasing capacity for cold acclimation (*a*); note that performance during cold and warm acclimation was measured in the same individuals, and the acclimation capacity was calculated across acclimation treatments (see text for more detail). However, performance breadth within acclimation treatments did not change with increasing capacity for cold acclimation (*b*). Within both cold and warm acclimation treatments, performance breadth decreased with increased maximal performance (*c*). Regression lines are shown for significant relationships.

Maximum performance during warm acclimation decreased with increasing acclimation capacity (*F*_1,26_=5.48, *p*<0.03, *R*^2^=0.17; figure 3*a*), but there was no significant relationship between maximal performance and acclimation capacity during cold acclimation (*F*_1,31_=1.46, *p*=0.24; figure 3*a*). Performance breadth did not change with acclimation capacity in either acclimation conditions (cold: *F*_1,31_=0.69, *p*=0.41; warm: *F*_1,26_=1.08, *p*=0.31; figure 3*b*). There was a negative relationship between maximum performance and performance breadth in fish within cold (*F*_1,31_=12.60, *p*=0.0012, *R*^2^=0.28) and warm (*F*_1,26_=4.62, *p*=0.041, *R*^2^=0.15) acclimation treatments (figure 3*c*), indicating a generalist–specialist trade-off in response to acute temperature changes.

## 5. Discussion

We show that there was pronounced variation between individuals in performance maxima, performance breadth and the capacity for acclimation. This is an important and interesting finding because it shows that the shape of performance curves is not characteristic of populations. The high variation between individuals indicates that there are genetic constraints within individuals that limit the shape and plasticity of reaction norms. There is considerable daily and seasonal variability in water temperature at the site of origin of the population from which we obtained our study animals [15]. Despite this environmental variability, selection has not reduced the variation in phenotypes, which indicates that there is no strong fitness differential between the different performance phenotypes [25].

As predicted, acclimation did result in a shift of the temperature at which maximal performance occurred. Some individuals had performance maxima above temperatures commonly experienced at the study site. Hence, unlike many terrestrial ectotherms [26] at least part of the population would be resilient to stochastic high-temperature events. This heat tolerance would render the species well suited to colonizing variable environments such as shallow and ephemeral water bodies, and it may partly explain why mosquitofish are such successful invaders [27]. On average, swimming performance was greater during warm acclimation than during cold acclimation, which would indicate that ‘warmer is better’ [28]. However, the pronounced difference between individuals limits the strength of this conclusion, particularly because differences between individuals were primarily caused by differences in their ability to acclimate. Additionally, we acclimated all fish to cool conditions first, and the possibility exists that there is an effect of the sequence of acclimation conditions on the thermal sensitivity of performance. Although such an effect was not observed in rainbow trout [12], it may be worthwhile testing in mosquitofish.

Contrary to our predictions, however, increased capacity for acclimation was not associated with decreased performance breath or increased maximal performance. We found the opposite to the latter prediction and show that cold acclimation comes at a cost, and performance maxima during warm acclimation decreased with increasing capacity for cold acclimation. Cold acclimation essentially represents a generalist strategy, and even in response to long-term changes in (acclimation) temperature there is a similar trade-off between performance maxima and breadth as that observed in response to acute change in temperature. There is a possibility that the time lag for warm acclimation is greater in animals that show greater capacity for cold compensation so that the trade-off could be weakened as the period of warm acclimation increases, and this transient acclimation response would be worth testing. Nonetheless, the trade-off represents a cost to cold acclimation and can explain the high variability in the capacity for acclimation between individuals.

The generalist–specialist trade-off appears to be a system property that manifests at the level of whole-animal performance in response to acute changes in temperature and during thermal acclimation. The trade-off is likely to be caused by underlying physiological traits that maximize performance at low temperatures but reduce it at high temperatures. Possible candidates could be the average fluidity of membranes which would increase activity of membrane-bound proteins at low temperature, but their lack of structural integrity at high temperatures may decrease enzyme activities [29,30]. Additionally, thermal acclimation can be mediated by regulators such as thyroid hormone that increases the concentrations of transcriptional regulators of metabolic genes, such as PGC-1*α*, which in turn leads to an increase in metabolic scope at low temperatures [11]. At the same time, thyroid hormone regulates skeletal muscle and heart function [31,32]. The end result of cold acclimation is that the physiological processes that enable locomotion—energy metabolism, skeletal muscle function and cardiovascular function—perform as well at low temperature as at high temperature as a result of thyroid-mediated changes in the concentrations of their functional components. Differences between individuals in their capacity for cold acclimation could be the result of differences in receptor densities that alter the sensitivity to regulatory mechanisms. Interestingly, there are indications that thyroid hormone can have opposite effects depending on whether animals are exposed to warm or cold conditions. For example, thyroid increases concentrations of transcripts of subunits of the metabolic enzymes cytochrome *c* oxidase and ATP synthase during cold exposure, but decreases them during warm exposure [11]. These opposite effects could result from the different modes of action of thyroid hormone [33] and could explain the trade-off we observed during acclimation. However, the reasoning above is speculative, and the physiological mechanisms that cause the trade-off are as yet unresolved. Clarifying the role of potential regulators such as thyroid hormones would provide important insights into the genetic mechanisms underlying performance curves.

## Supplementary Material

Supplementary Data: the complete dataset associated with this ms.

## Data accessibility

All data are attached as electronic supplementary material.
